# Grand Challenges in Oral Cancers

**DOI:** 10.3389/froh.2020.00003

**Published:** 2020-06-09

**Authors:** Ricardo D. Coletta, W. Andrew Yeudall, Tuula Salo

**Affiliations:** ^1^Department of Oral Diagnosis, School of Dentistry, University of Campinas, São Paulo, Brazil; ^2^Department of Oral Biology & Diagnostic Sciences, The Dental College of Georgia at Augusta University, Augusta, GA, United States; ^3^Cancer and Translational Medicine Research Unit, Faculty of Medicine and Medical Research Center Oulu, Oulu University Hospital, University of Oulu, Oulu, Finland; ^4^HUSLAB, Department of Pathology, Institute of Oral and Maxillofacial Disease, University of Helsinki, Helsinki University Hospital, Helsinki, Finland

**Keywords:** oral squamous cell carcinoma, younger age group, biomakers, treatment, prognosis

Oral squamous cell carcinomas (OSCC), arising from surface epithelium, constitute more than 90% of all oral cancers and, in many studies, the term oral cancer is applied as synonymous of OSCC. OSCC, together with SCC from pharynx, larynx, nasal cavity, and paranasal sinuses, belongs to the group of head and neck SCC, but due to increasing knowledge of specific risk factors, different genetic mutations and epigenetic changes and, more importantly, distinct biological and clinical behaviors, tumors from those different sites must be studied separately. Other neoplasms may also arise in the oral cavity, including those derived from connective tissues, minor salivary glands, lymphoid tissues, melanocytes, and odontogenic apparatus, as well as metastasis from distant tumors. Due to their relatively low incidences, important areas of future research must include etiopathogenetic mechanisms, features allowing differential diagnosis, therapeutic strategies (particularly target-specific interventions), and prognostic markers.

OSCC is one of the most prevalent cancers worldwide, with a global incidence of more than 350,000 new cases and 177,000 deaths every year, though with considerable geographic and environmental risk factor differences [1]. The incidence of OSCC has been decreasing in some areas of the world, but the incidence has risen in some countries (mainly low-income countries) and among females [2, 3]. An alarming increase in the incidence of OSCC in the younger age group (≤45 years-old) has also been observed. While the use of all forms of tobacco and alcohol explains this increasing incidence in some countries and among females, as ~80% of the world's smokers live in developing countries and females are more exposed to tobacco and alcohol nowadays than before, they do not explain the incidence among younger cancer patients, who, in most of the cases, lack those traditional risk factors or, when present, the time of exposure is much shorter. In this sense, some specific issues regarding OSCC in the younger age group need to be addressed, including risk factors, patterns of inheritance of predisposing genetic alterations, clinical behavior, and prognosis. Furthermore, more effective programs, particularly in developing countries, to eliminate or reduce tobacco (smoking and chewing) and alcohol consumption would be of great value for reducing the incidence of OSCC and other cancers related to those traditional risk factors.

Oral potentially malignant disorders (OPMD), mainly represented by leukoplakia, erythroplakia, oral submucous fibrosis, and proliferative verrucous leukoplakia (PVL), are well-recognized to precede the development of OSCC. In this group, PVL seems unique, since it does not consistently show association with classical environmental agents, its natural history seems different than any other OPMD, and the potential of malignant transformation is the highest among OPMD [4]. The potential of other OPMD, such as oral lichen planus (OLP), remains still somewhat questionable. However, several meta-analyses published in the last 3 years showed a low, but consistent, transformation of classical OLP to oral cancer, confirming that OLP should be considered as an OPMD [5–7]. The early diagnosis and treatment of OPMD is essential to minimize or even eliminate the risk of malignant transformation. However, not all disorders are amenable to curative treatment, and the transformation does not occur in every single case. Although presence and intensity of dysplasia, representing the collection of changes in cellular morphology and tissue architecture at the histopathological standpoint, are considered the main parameters related to malignant transformation of OPMD, the histological assessment of the epithelial dysplasia is a source of substantial subjectivity, and in a meta-analysis, the mean overall transformation rate of OPMD with dysplasia was only 12.1% [8]. Therefore, the characterization of biomarkers to define the magnitude of risk, the mechanisms and the period of progression for transformation is of great importance, in order to rationally schedule treatment or follow-up and to plan cost-effective oral screening programs.

OSCC is considered a very aggressive tumor and the majority of patients displays a locoregionally advanced disease at diagnosis, for which multimodality therapy is required. Tumor invasion, lymph node metastasis and high rates of locoregional recurrence, besides development of second primary tumors, are the leading causes of death in OSCC patient. However, even at early stage, particularly tumors of the tongue and floor of mouth may be very aggressive, with increased tendency to invasion and metastasis. In this context, survival rates are of ~40–50% and these rates have not significantly changed over the past decades [9]. Although our understanding about the biological processes involved in cancer development and progression is evolving and many biomarkers have been suggested to significantly impact diagnosis and prognosis of OSCC, no biomarker has yet met the stringent criteria that are needed to be used in clinical practice.

Thereafter, it is of consensus among all professionals involved in the field that markers with potential clinical applications, such as early diagnosis, therapeutic targets, responsiveness to treatment, prognosis and post-therapeutic monitoring, are urgently needed to improve clinical management of OSCC. Investigations aimed to characterize OSCC biomarkers should ideally take into consideration some aspects, including prospective analyses of several markers, as a panel, in large cohorts, preferably multicenter, and application of quantitative and complementary assays to capture the impact of biomarkers in different scenarios (the vast majority of studies is based exclusively on immunohistochemistry without a standard pattern of quantification). Furthermore, authors should be aware that cancer heterogeneity is an important drawback for predictive biomarkers. Cancer generally starts as a monoclonal disease but, with its evolution, heterogeneous populations of tumor cells arise. Moreover, in the oral cavity, field cancerization is a factor that is likely to contribute to development of multiple primary tumors within the field of exposure, perhaps contributing further to heterogeneity of clinical disease. Patients harboring the same OSCC, such as location, clinical stage, and histopathological features, may contain cells with distinct patterns of mutations and epigenetic alterations, which may influence the biological behavior and result in significantly different clinical outcomes. In this setting, markers and therapeutic targets against cancer stem cells, which are drivers of self-renewal, metastasis, relapse and resistance to conventional therapy, hold great potential.

The identification of genetic variants of risk, mutated genes, activated pathways and networks, and the characterization of potential coadjutants of the tumor cells such as the components of tumor microenvironment are of utmost importance to provide preclinical data to support clinically relevant advances. Indeed, the era of “omics,” such as genomics, epigenomics, transcriptomics, proteomics, metabolomics, and some others including genome-wide analysis of genetic variants, which are based on large-scale datasets, is allowing important advances to characterize the functional role of specific genes through their RNAs (including regulators such as non-coding RNAs) and proteins and to identify candidate therapeutic targets for some tumors. Many genome-wide profiling studies with OSCC samples are emerging, and it is time now to transform those promising data highlighting biomarkers and targeted therapies to change the dark scenario of survival rates. Liquid biopsy, the analysis of non-solid biological samples, particularly blood and saliva, has rapidly gained great attention as a potential tool for identification of cancer biomarkers. In those easily accessible body fluids, circulating tumor cells, tumor DNA, RNA, or specific proteins, and extracellular vesicles can be assessed. Although the potential is enormous, especially for OSCC-screening programs, we need to define the biomarker(s) in those fluids and to design clinical validation studies with long-term outcomes.

Recent evidence suggests a link between chronic inflammatory disease, such as periodontitis, and oral cancer, supporting the longstanding concept that poor oral hygiene may underpin oral carcinogenesis. As the oral cavity is a reservoir for many hundreds of species of bacteria, one or more of these organisms may contribute to tumor development or progression. To date, bacteria that are associated with periodontal disease, including *Prevotella intermedia, Porphyromonas gingivalis*, and *Fusobacterium nucleatum*, have been associated with oral cancer [10, 11], although definitive mechanistic links are currently lacking. One possibility may be the ability of some oral bacteria to trigger host intracellular signaling pathways that lead to production of chemokines that are then able to deregulate host epithelial cell growth, as well as modifying the immune cell infiltrate in the local microenvironment. Therefore, understanding the role of the oral microbiome in the genesis of oral cancers is a key challenge for researchers, the answer to which might even help to explain the observations that, increasingly, oral cancers are being identified more frequently in patients lacking the traditional risk factors of tobacco and alcohol use, as mentioned above.

Surgery remains the preferred treatment for OSCCs, with adjuvant radiotherapy with or without chemotherapy in cases at advanced stage. However, immunotherapy and targeted-therapy are showing very promising results. For example, the use of PD-1 and PD-L1 immune checkpoint inhibitors for treatment of recurrent/metastatic cancer has been tested in phase III randomized clinical trials, and it is now suggested that these inhibitors should be used to manage otherwise intractable lesions [12]. PD-1 inhibitors have been shown a superior performance in patients whose disease is refractory to platinum chemotherapy, prolonging survival. Moreover, monotherapy with pembrolizumab (anti-PD-1 humanized antibody) is approved to treat recurrent/metastatic cancers that overexpress PD-L1, a PD-1 receptor ligand. Comprehensive understanding of the molecular events that drive oral tumorigenesis has provided rationale for the design and implementation of targeted therapeutic strategies to combat malignant progression [13]. Examples include epidermal growth factor receptor (EGFR)-driven mechanisms, to which monoclonal antibodies and small molecule inhibitors are available and in use. However, major roadblocks exist, such as the lack of a robust clinical response in many cases, due at least in part to the rapid development of drug-resistant phenotypes. Furthermore, components of tumor microenvironment have shown potential role as biomarkers and an interesting alternative to traditional tumor cell-directed therapy. A major challenge for the oral cancer scientific community will be to identify and overcome these therapeutic shortfalls.

The efficiency of treatments for oral cancer is not constant, whereby responses vary from patient to patient. This can cause several major problems including unnecessary side effects, overtreatment, worsening prognosis and increasing treatment costs. Therefore, an urgent need exists to develop efficient and reliable methods to test the efficacy of cancer drugs and irradiation before administered to patients. Similar to other cancer types, several *in vitro* and *in vivo* models are suggested for testing cancer drugs and irradiation on OSCC patient samples, however, the vast majority of these models does not have validation with a head-to-head comparison of patient response, making their clinical translation difficult at present. As the field of personalized medicine is rapidly growing, personalized approaches for OSCC treatment must be prioritized.

OSCC patients may experience a wide range of oral dysfunctions during treatment, with some of those lasting for the live. The major sources of these dysfunctions are the surgeries and side effects of adjuvant therapy. Although many studies are focused in determining factors associated with those dysfunctions, preventive strategies and treatment options remain challenges to be overcome.

The complexity of today's scientific and professional world, the fast pace of knowledge acquisition, and the nature of health problems yet to be solved or adequately addressed, are such that a team approach, involving different talents with complementary knowledge, backgrounds and skills, is required. In many cases this is not only true for the advancement of relevant knowledge, but also for the application of existing knowledge in the health care setting. The team approach allows both depth in the knowledge and stimulation of research, which are essential to our fight against oral cancer. We are glad to release the Oral Cancers section of Frontiers in Oral Health—a multidisciplinary forum for publications in those important areas of oral cancer, from basic research to clinical practice, for translation of scientific advances into novel diagnostic, therapeutic, and prognostic approaches to improve patient's outcome. This section has associate and reviewing editors with different backgrounds, who are real experts in their fields.

## Author Contributions

All authors listed have made a substantial, direct and intellectual contribution to the work, and approved it for publication.

## Conflict of Interest

The authors declare that the research was conducted in the absence of any commercial or financial relationships that could be construed as a potential conflict of interest.
